# Senior High School Students' Knowledge and Attitudes Toward Information on Their Health in the Kumasi Metropolis

**DOI:** 10.3389/fpubh.2021.752195

**Published:** 2022-01-13

**Authors:** Simon Boateng, Akosua Baah, Doris Boakye-Ansah, Bosco Aboagye

**Affiliations:** ^1^Social Sciences Department, St. Monica's College of Education, Mampong, Ghana; ^2^Mampong Technical College of Education, Mampong, Ghana

**Keywords:** senior high school, Kumasi Metropolis, malaria, cholera, information on health

## Abstract

The study examines senior high school students' understanding and attitudes toward information on their health in the Kumasi Metropolis. Multiple sampling techniques (convenient and simple random sampling techniques) were used in the study. A questionnaire was used to collect data from 391 respondents for the study. Frequencies and percentages were used to analyze the sociodemographic data. Again, the study used Pearson's correlation coefficient to show the degree of relationship between the level of knowledge of health information and attitudes toward seeking and sharing health information. The study found students' knowledge of the causes and symptoms of malaria, cholera, and Sexually Transmitted Infections (STIs) to be appreciably high as a result of readings from textbooks and health professionals. Again, the study found that the students preferred sharing their health information with friends than their parents and schools' authorities. The study further found that the major sources of students' health information included health professionals and textbooks. Lastly, even though some of the students claimed internet sources to their health information, it was not a major source to the student body at large. The study recommends strong health systems on the campuses of senior high schools as they have become communities on their own as a result of the emergence of the free senior high school program. The monitored positive peer-counseling group should also be encouraged by the schools' management and by extension the counseling units for the students to share views on themselves, particularly on health issues where they deem fit.

## Introduction

Information about health is very important if society wants its citizens to stay healthy. Information on health is data collected regarding a person's medical history that include signs and symptoms of disease, diagnoses, medical procedures, and outcomes (1, 2). To ensure improved information on people's health, a health information system is introduced (Chen et al., 2018) (3). The goal of health information systems is to improve treatment for patients by having the most current patient's data available to every healthcare practitioner who treats this client (3, 4). However, health information system is available only at healthcare facilities and accessed and used by health professionals for treatment.

It is, however, imperative to note that a patient's information on health can be viewed in two ways; either gathered individually or through a gathered data set of the population (5, 6). Apart from information on health gotten directly from physicians, nurses, pharmacists, and other health practitioners, it can be gotten from the internet, textbooks, and newsletters as well (7, 8). However, not all the sources of information on health are credible. For instance, Hampton (8) indicated that people facing medical decisions often look beyond their physicians to social media, websites, videos, and mobile applications which do not proffer the needed remedy. It is further explained that these people who most often seek information on health from the internet have not experienced any positive change in their lives (8–10). This was confirmed by Keselman et al. (11) that most information on health on the internet is poor and not credible leading to more harm than good.

On the other hand, seeking information on health among the populace has brought some changes in the lives of people toward medical use and lifestyle due to increased understanding of health issues (12–14). McNicol (15) and Dutta-Bergman (16) explained that the current increase in consumer freedom to act in health care accompanied by the use of social media, websites, and mobile applications for information on health gathering has led to increasing interest of understanding the consumer health information. Osei-Assibey et al. (17) further noted that this act has led to increased adherence to medical prescriptions.

That notwithstanding, the youth have been deemed to be more vulnerable to information on health sourced from the social media and websites (18, 19). Valle et al. further explained that senior high school students enter a critical transition and begin to become independent and responsible for their own health during high school years. Moreover, high school students are thought to be a vulnerable population in that they are exposed to all kinds of health risks (20, 21). Common health risk among senior high school students (especially those in the boarding houses) includes skin rashes due to congestion in dormitory, malaria, and cholera due to unsanitary environment, and sexually transmitted diseases due to indiscriminate sex (22).

In view of this, more health education programs are organized from time to time by government agencies and non-governmental organizations for the students. The programs most often center on current diseases and infirmities and diseases that are more prevalent (13, 17, 23–25). However, despite these attempts, communicable diseases are prevalent in the senior high schools in the Kumasi Metropolis. This has raised concerns about students' understanding and attitudes toward seeking information on their health.

Again, despite the various attempts to increase knowledge on health among the students, understanding and attitudes among students toward some health issues are not positive. Ezeala-Adilkaibe et al. (26) revealed in South East Nigeria that the majority of students have poor knowledge, attitude, and practice of epilepsy. Brass et al. (27) added that students' knowledge about HIV or AIDS is inadequate and their attitudes were stigmatized. Meanwhile, Thanavanh et al. (2) noted that students with medium- and high-level knowledge, attitudes, and practices regarding HIV or AIDS are likely to display a positive attitude toward people living with HIV.

In view of the importance of information on health among students, there have been several attempts to help senior high school students get more oriented with the information on their health. Thus, this study examines senior high school students' understanding and attitudes toward information on their health in the Kumasi Metropolis.

## Materials and Methods

### Research Design

This study was a cross-sectional study where primary data were collected from students in selected senior high schools in the Kumasi Metropolis. Based on the formulated research questions that the researchers wanted to answer and the nature of the study, the quantitative research design was most appropriate, hence its adoption in this study. The quantitative design helped the researcher to estimate the relationships between the variables understudy. Thus, in this study, the quantitative research design helped the researcher to determine the relationship among the level of knowledge and seeking and sharing health information among the students.

### Study Population and Sampling Procedure

Students in senior high schools in the Kumasi Metropolis were the target population. The metropolis has a number of senior high schools, both near and far from the researchers. This study employed convenience sampling to sample the schools and students (28). The schools were selected based on proximity and the willingness of the schools' administration and individual students to participate. Based on these two criteria, five schools were sampled from the study area. The study considered proximity to be how the schools were closer to each other and to the researchers as well. The researchers choose 5 out of 21 senior high schools in the metropolis based on the assumption of similar characteristics of the students. The age range of senior high school students was between 15 and 18 years. This connotes homogenous adolescent characteristics for these students understudy; hence, the study's generalization is justified. The study admits that the heterogeneous socioeconomic background of students might have caused different perspectives about the problem understudy. However, since the students were all in the same schools facing virtually the same problems, such differences were not significant to be noticed.

Again, premised on the principle of anonymity and ethical consideration, the names of the schools have been withheld and classified them as Schools A, B, C, D, and E. It is imperative to note that Schools A and B were strictly boys' schools, C and D were strictly girls' schools whereas E was a mixed school (both boys and girls). These schools were chosen because they are considered as the elite schools with most of the best educational facilities for academic work in the study area. Again, as stated earlier, the researchers assumed that since the students were in their teens, their perspectives about their health information would not differ much. Based on the aforementioned assumptions, the schools were chosen for the study. The respective population of the sampled schools are shown in Table 1.

**Table 1:** Student population of sampled schools.

**Schools**	**Student population**
School A	3,516
School B	3,267
School C	3,154
School D	3,599
School E	2,987
**Total**	**16,523**

Aside the study sampling of the schools, the study sampled the students through convenience sampling. The criteria used here were “easy access to students” (29). This study realized four places where students could be found easily. First, in classrooms during the lesson; second, at dining hall during dining time; third, at the canteen during break time, and fourth, at school entrance both in the morning and after school. Among these four places, canteen during break time and at the entrance of dining hall after dining periods were most convenient due to time and activities of the students. The assumption for choosing these places was that at least, a student would be found at one of the venues at the time of data collection. The researchers first sought the consent of participants and assured them of strict confidentiality. The researchers then read and explained the questions to the participants before answers were required. Because the researchers had a fair knowledge about the exact number of sample size they were seeking to sample, they did not continue the data collection after accurately getting the exact number (391).

### Sample Size Determination

The study employed Yamane (30) sample size determination formula in Equation (1) to compute the overall size for the study.


(1) $$\begin{array}{r} n=\frac{N}{1+Ne^{2}} \end{array}$$

where: N signifies the population under study = 16523

e signifies the margin of error = 0.5

n signifies the sample size = 391

From the formula in Equation (1);


$$n=\frac{16523}{1+16523\;{\left(0.05\right)}^{2}}=390.5454116=391$$

Therefore, the overall sample size for this study was 391. This study further used a proportionate stratified population sampling technique to determine the sample size for each school as shown in Table 2.

**Table 2:** Sample size for each student population of sampled schools.

**Schools**	**Student population**	**Relative reference**	Sample size Rf*391
School A	3,516	0.2128	83
School B	3,267	0.1977	77
School C	3,154	0.1909	75
School D	3,599	0.2178	85
School E	2,987	0.1808	71
**Total**	**16,523**	**1.0000**	**391**

### Data Collection Instrument

Among the data collection instruments (questionnaire and interview guide), this study relied on the questionnaire to collect all primary data from the students. This study used a structured questionnaire because it helped the researcher to collect standardized data, and second to collect data at a cheaper administration cost (31, 32). The questionnaire design has four sections. Section I focused on demographic data; Section II focused on the level of knowledge of health information; Section III focused on attitudes toward seeking health information; and Section IV on the attitudes toward sharing health information. All the questions contained in the questionnaire, except those under Section I (demographic data), were in the form of a five-point Likert scale.

### Data Collection Procedures

This study collected all primary data between December 9, and December 13, 2020. The study sought permission from the headmasters or headmistresses of the selected senior high schools through an introductory letter obtained from St. Monica's College of Education. The researchers administered questionnaires in the selected schools by themselves. A strategy was devised to ensure a high response rate. This was achieved by encouraging all respondents to fill in the questionnaire in the presence of the researchers. This did not only ensure a high response rate but also offered the opportunity to clarify all misunderstandings surrounding some of the research questions.

The participation in the study was not compulsory but students willingly participated and gave out accurate data (33, 34). The study informed the purpose of the study to all respondents and assured them strict confidentiality and anonymity. Before the actual data collection, this study ensured that the instrument used is valid and reliable. The researchers showed the research instruments to their colleagues who helped to restructure the questionnaire to be more consistent with the research objectives. For reliability, the researchers pre-tested the questionnaire at SIMMS Senior High School in the Kwabre East Municipality on 25 students. This was performed to ensure that errors in the questionnaire were corrected before actual administration.

### Data Analysis

The data collected were analyzed with the use of descriptive and inferential statistics. The data were cleaned and entered into Statistical Package for Social Scientists (SPSS) version 21.0. For the descriptive analysis, frequencies and percentages were used to analyze the data. For the inferential analysis, this study used Pearson's correlation coefficient to show the degree of relationship among the level of knowledge of health information and attitudes toward seeking and sharing information on health. In furtherance, data were presented as numbers and percentages for categorical variables.

### Ethical Approval

The study was approved by the Committee on Human Research, Publication, and Ethics of the School of Medical Sciences, Kwame Nkrumah University of Science and Technology/Komfo Anokye Teaching Hospital with reference number CHRPE/AP/317/20. Again, all participants gave verbal consent for their participation in the study.

## Results and Discussion

This section presents and discusses data collected from 391 students in Kumasi Metropolis concerning health information through questionnaire administration. The presentations and discussions of data were in accordance with the arrangement of research questions. The sociodemographic characteristics of the respondents were first presented and discussed to form the basis of discussions in this study.

### Sociodemographic Characteristics of Respondents

This section talks about the sociodemographic characteristics of the respondents. These characteristics included sex, class, and program of study of students understudy. The sociodemographic characteristics of the respondents (students) are shown in Table 3.

**Table 3:** Sociodemographic characteristics of respondents (*N* = 391).

**Variables**	**Category**	**Frequency**	**Percentage**
Sex	Boy	213	54.5
	Girl	178	45.5
Class	Form 2	165	42.2
	Form 3	226	57.8
Program of study	Home economics	90	23.0
	Business	75	19.2
	General arts	100	25.6
	Visual arts	80	20.5
	Science	46	11.7

*Source: Field data (2021)*.

From Table 3, out of 391 respondents, 213 (54.5%) were boys whereas 178 (45.5) were girls. Even though gender parity at the senior high schools has been attained in Ghana, male students dominated in the study as against their female counterparts. This may be attributed to the willingness of the male students to take part in the study at the time of data collection. The class distribution of the respondents is shown in Table 3 as 165 (42.2%) for Form 2 and the remaining 226 (57.8%) for Form 3. The number of the Form three (3) students increased because, at the time of the data collection, all the Form 3 students were in school. However, only the gold track Form one and two students were present. This explained why the number of students from Form two was relatively lower compared with the Form three students. The students from form one were not considered since they were new to the schools; hence, any information from them may be insignificant since it would not be a true reflection of information on their health and usage behavior of the students on campus.

In relation to program of study in senior high schools in Kumasi Metropolis, Table 3 shows that 90 (23.0%) of the respondents offered home economics, 75 (19.2%) offered business, 100 (25.6%) offered general arts, 80 (20.5%) offered visual arts, and 46 (11.7%) offered science. The study shows that majority of the students offered general arts and home economics. This may be because general arts and home economic courses are now the mostly considered courses in the admission into nurses training colleges and colleges of education in Ghana. As a result, most students who want to pursue nursing end up pursuing general arts and home economics. Moreover, most of the students have the notion that general arts and home economics are easy to pass and this influences most of the students to offer both.

### Level of Knowledge on the Sign and Symptoms of Common Diseases

The study sought the respondents' knowledge on the symptoms and causes of some common diseases (malaria, cholera, and STIs) among the students. The responses were collected from the questionnaire and summarized in Table 4.

**Table 4:** Level of knowledge on the sign and symptoms of common diseases.

**Statement**	**Low**	**High**	**Very high**
Causes of malaria	76 (19.4%)	125 (32.0%)	190 (48.6%)
Symptoms of malaria	74 (18.9%)	140 (35.8%)	177 (45.3%)
Causes of cholera	90 (23.0%)	135 (34.5%)	166 (42.5%)
Symptoms of cholera	85 (21.7%)	145 (37.1%)	161 (41.2%)
Causes of STIs	78 (19.9%)	165 (42.2%)	148 (37.9%)
Symptoms of STIs	88 (22.5%)	158 (40.4%)	145 (37.1%)

*Source: Field data (2021)*.

From Table 4, out of 391 respondents, 76 (19.4%) noted a low level of knowledge, 125 (32.0%) expressed a high level of knowledge, and 190 (48.6%) indicated a very high level of knowledge on the cause of malaria among students in the Kumasi Metropolis. The study shows that most of the students in Kumasi Metropolis have a high level of knowledge on the causes of malaria. This is because malaria is one of the common diseases that affect most of the students in the study schools. In addition, Table 4 further shows that 74 (18.9%) of the respondents expressed a low level of knowledge, 140 (35.8%) noted a high level of knowledge, and 177 (45.3%) indicated a very high level of knowledge on the symptoms of malaria. Thus, the study revealed that the majority of the respondents had a high level of knowledge on the symptoms of malaria. The respondents gave some of the symptoms of malaria to include feeling hot and shivery, headaches, vomiting, muscle pains, diarrhea, and generally feeling unwell. They however added that some of the symptoms are often mild and can sometimes be difficult to identify as malaria.

Again, Table 4 shows that 90 (23.0%), 135 (34.5%), and 166 (42.5%) expressed low, high, and very high levels of knowledge on the cause of cholera, respectively. The study shows that majority of students in Kumasi Metropolis have a high level of knowledge on the causes of cholera. This may be as a result of the fact that most of the students asserted, that they had suffered from the disease since they came to the school. The results in Table 4 reveal that 85 (21.7%) expressed a low level of knowledge, 145 (37.1%) showed a high level of knowledge, and 161 (41.2%) indicated a very high level of knowledge on the symptoms of cholera among students in senior high schools in Kumasi Metropolis. The results show that majority of the students have more information and knowledge about the symptom of cholera.

Moreover, from Table 4, out of 391 respondents, 78 (19.9%) noted a low level of knowledge, 165 (42.2%) indicated a high level of knowledge, and 148 (37.9%) expressed a very high level of knowledge about the causes of sexually transmitted infections (gonorrhea and syphilis). The study revealed that students in senior high schools in the Kumasi Metropolis have a high level of knowledge on the causes of STIs (gonorrhea and syphilis). Table 4 shows that 88 (22.5%) noted a low level of knowledge, 158 (40.4%) indicated a high level of knowledge, and 145 (37.1%) expressed a very high level of knowledge on the symptoms of STIs in the Kumasi Metropolis. The study revealed that most of the students in Kumasi Metropolis know the symptom of STIs. They however could not give more important symptoms of STIs.

### Attitudes Toward Seeking Health Information

The section further sought to determine the attitudes of students toward seeking information on their health in the Kumasi Metropolis. The study asked the respondents to indicate how often they use any of the following sources to seek information on their health. The responses were collected from the questionnaire and summarized in Table 5.

**Table 5:** Attitudes toward seeking health information (*N* = 391).

**Statements**	**Rarely**	**Not often**	**Often**	**Very often**	**Extremely often**
Internet	90 (23.0%)	145 (37.1)	75 (19.2%)	43 (11.0%)	38 (9.7%)
Health professionals	34 (8.7%)	54 (13.8%)	48 (12.3%)	130 (33.2%)	125 (32.0%)
Friends	39 (10.0%)	42 (10.7%)	20 (5.1%)	120 (30.7%)	170 (43.5%)
Textbook	37 (9.5%)	32 (8.1%)	30 (7.7%)	168 (43.0%)	124 (31.7%)
Newsletters	165 (42.2)	80 (20.4%)	50 (12.8%)	55 (14.1%)	41 (10.5%)

*Source: Field data (2021)*.

Results from Table 5 show that 90 (23.0%) noted rarely, 145 (37.1%) not often, 75 (19.2%) often, 43 (11.0%) very often, and 38 (9.7%) expressed extremely often that they seek information on their health using the internet. This shows that most of the students in Kumasi Metropolis do not use the internet to seek information on their health. Again, from Table 5, the results show that 34 (8.7%), 54 (13.8%), 48 (12.3%), 130 (33.2%), and 125 (32.0%) noted rarely, not often, often, very often, and extremely often, respectively, that they seek information on their health from health professionals. This clearly shows that the majority (77.5%) of the respondents often seek information on their health from the health professionals.

Moreover, results in Table 5 show that 39 (10.0%) indicated rarely, 42 (10.7%) not often, 20 (5.1%) often, 120 (30.7%) very often, and 170 (43.5%) extremely often seek information on their health from their friends. This suggests that the majority (79.3%) of the respondents asserted that they seek information on their health from their friends. In addition, from Table 5, 37 (9.5%), 32 (8.1%), 30 (7.7%), 168 (43.0%), and 124 (31.7) of the respondents noted rarely, not often, often, very often, and extremely often, respectively, that they seek information on their health from textbooks. The study reveals that most of the students in Kumasi Metropolis seek information on their health from their textbooks.

Last but not least, Table 5 shows that 165 (42.2%) noted rarely, 80 (20.4%) not often, 50 (12.8%) often, 55 (14.1%) very often, and 41 (10.5%) extremely often that they seek information on their health from newsletters. This suggests that the students in Kumasi Metropolis do not seek health information from newsletters.

### Association Between Information Sources and Level of Health Knowledge

This study used the chi-square test to test for a statistical association between each source of information on their health and the level of knowledge on the same. The results have been illustrated in Table 6.

**Table 6:** Association between level of knowledge and sources of information on health.

**Variable**	**Internet source**	**Health professional source**	**Friends source**	**Textbook source**	**Newsletter source**
Level of knowledge on causes of malaria	15.456 (0.014)*	20.354 (0.001)**	18.867 (0.001)**	19.578 (0.001)**	19.856 (0.001)**
Level of knowledge on symptoms of malaria	13.322 (0.012)*	19.689 (0.001)**	11.256 (0.007)*	18.795 (0.001)**	16.845 (0.001)**
Level of knowledge on causes of cholera	14.482 (0.022)*	21.856 (0.001)**	9.853 (0.068)	14.533(0.022)*	6.789 (0.145)
Level of knowledge on symptoms of cholera	14.589 (0.021)*	19.858 (0.001)**	8.256 (0.078)	13.442 (0.032)*	9.856 (0.098)
Level of knowledge on causes of STIs	22.259 (0.001)**	23.523 (0.001)**	5.532 (0.132)	18.120 (0.001)**	9.258 (0.096)
Level of knowledge on symptoms of STIs	20.985 (0.001)**	19.885 (0.001)**	6.927 (0.115)	16.524 (0.024)	8.789 (0.159)

Source: Field data (2021); figures outside the parentheses are chi-square values; figures in the parentheses are p-values; **significant at 1%; and *significant at 5%.

From Table 6, the study found a significant association between the level of knowledge of the causes of malaria and seeking health information from the internet (chi-square value = 15.456, *p* = 0.014), health professional (chi-square value = 20.354, *p* < 0.001), friends (chi-square value = 18.867; *p* < 0.001), and textbooks (chi-square value = 19.578, *p* < 0.001). That is, as the students seek information on their health from the internet, health professionals, friends, and textbooks, their level of knowledge on the causes of malaria is increased.

Further, Table 6 shows that there is significant association between the level of knowledge on the symptoms of malaria and seeking information on their health from the internet (chi-square value = 13.322, *p* = 0.012), health professionals (chi-square value = 19.689, *p* < 0.001), and textbooks (chi-square value = 18.795, *p* < 0.001) at 5% significant level. This shows that students' level of knowledge on the symptoms of malaria increases when they seek information on their health from the internet, health professional, and textbooks.

The analysis in Table 6 shows that there is significant association between the level of knowledge on the causes of cholera and seeking information on their health from the internet (chi-square value = 14.482, *p* = 0.022), health professional (chi-square value = 21.856; *p* < 0.001), and textbooks (chi-square value =14.533, *p* = 0.022). This shows that students' level of knowledge on the causes of cholera increases when they seek information on their health from the internet, health professional, and textbooks. Similarly, the level of knowledge on symptoms of cholera was significantly associated with seeking information on health from the internet, health professionals, and textbooks. Again, from Table 6, there is a significant association between the level of knowledge of the causes of STIs and seeking information on their health from the internet (chi-square value = 22.259, *p* < 0.001), health professional (chi-square value = 23.523, *p* < 0.001), and textbooks (chi-square value = 18.120, *p* < 0.001). That is, the more the students seek information on their health from the internet, health professional, and textbooks, the higher their level of knowledge on the causes of STIs. Similarly, the level of knowledge on symptoms of STIs is significantly associated with seeking information on health from the internet, health professionals, textbooks, and newsletters.

### Attitudes Toward Sharing Health Information

Again, this section sought to know the attitudes of students toward sharing information on their health. The researchers asked the respondents to indicate the extent of their agreement or disagreement with each of the following statements. The responses were collected from the questionnaire and summarized in Table 7. This study further used the chi-square to test the significance of association between attitudes toward sharing information on their health and the characteristics of the respondents.

**Table 7:** Attitudes toward sharing health information by students.

**Variable**	**Characteristics of students**	**Category**	**SD**	**D**	**N**	**A**	**SA**	**Total**	**Chi-square value**	***p*-value**
Sharing information on health with friends	Sex	Boy	18 (42.9)	22 (57.9)	10 (40.0)	58 (31.0%)	37 (36.3)	213 (54.5)	17.285	0.001
		Girl	24 (57.1)	16 (42.1)	15 (60.0)	126 (68.5%)	65 (63.7)	178 (45.5)		
		**Total**	**42**	**38**	**25**	**184**	**102**	**391**		
	Class	Form 2	16 (38.1)	18 (47.4)	14 (56.0)	65 (35.3)	52 (51.0)	165 (42.2)	19.258	0.001
		Form 3	26 (61.9)	20 (52.6)	11 (44.0)	119 (64.7)	50 (49.0)	226 (57.8)		
		**Total**	**42**	**38**	**25**	**184**	**102**	**391**		
Sharing information on health with parents	Sex	Boy	60 (57.1)	32 (55.2)	18 (32.7)	75 (60.0)	28 (58.3)	213 (54.5)	13.285	0.004
		Girl	45 (42.9)	26 (44.8)	37 (67.3)	50 (40.0)	20 (41.7)	178 (45.5)		
		**Total**	**105**	**58**	**55**	**125**	**48**	**391 (100.0)**		
	Class	Form 2	30 (28.6%)	24 (41.4)	14 (25.5)	68 (54.4)	29 (60.4)	165 (42.2)	9.527	0.069
		Form 3	75 (71.4)	34 (58.6)	41 (75.5)	57 (45.6)	19 (39.6)	226 (57.8)		
		**Total**	**105**	**58**	**55**	**125**	**48**	**391 (100.0)**		
Sharing information on health with health professionals in the schools	Sex	Boy	18 (40.9)	22 (44.9)	16 (42.1)	75 (51.7)	82 (71.3)	213 (54.5)	17.987	0.015
		Girl	26 (59.1)	27 (51.1)	22 (57.9)	70 (48.3)	33 (28.7)	178 (45.5)		
		**Total**	**44**	**49**	**38**	**145**	**115**	**391 (100.0)**		
	Class	Form 2	16 (36.4)	20 (40.8)	14 (36.8)	60 (41.4)	55 (47.8)	165 (42.2)	19.527	0.001
		Form 3	28 (63.6)	29 (59.1)	24 (63.2)	85 (58.6)	60 (52.2)	226 (57.8)		
		**Total**	**44**	**49**	**38**	**145**	**115**	**391 (100.0)**		
Sharing information on health with school authorities	Sex	Boy	18 (56.3)	32 (61.5)	24 (57.1)	85 (54.8)	54 (49.1)	213 (54.5)	16.231	0.023
		Girl	14 (43.8)	20 (38.5)	18 (42.9)	70 (45.2)	56 (50.9)	178 (45.5)		
		**Total**	**32**	**52**	**42**	**155**	**110**	**391 (100.0)**		
	Class	Form 2	17 (53.1)	17 (32.7)	19 (44.2)	69 (44.5)	43 (39.1)	165 (42.2)	18.258	0.001
		Form 3	15 (46.9)	35 (67.3)	23 (54.8)	86 (55.5)	67 (60.9)	226 (57.8)		
		**Total**	**32**	**52**	**42**	**155**	**110**	**391 (100.0)**		

*Source: Field data (2021); where SD, strongly disagree; D, disagree; N, neutral; A, agree; SA, strongly agree; frequencies are outside the parentheses and percentages inside the parentheses (5% significant level)*.

From Table 7, out of 42 respondents who noted strongly disagree that they share information on their health with friends, 18 (42.9%) were boys and 24 (57.1%) were girls. From Table 7, out of 38 respondents disagreed that they share information on their health with friends and of out this number, 22 (57.9%) were boys and 16 (42.1) were girls. Furthermore, out of 391 respondents, 25 of the respondents who expressed neutral that they share information on their health with friends 10 (40.0%) and 15 (60.0%) were boys and girls, respectively. Among the 184 of the respondents who agreed that they share information on their health with friends, 58 (31.5) were boys and 126 (68.5%) were girls. In addition, out of 102 respondents who noted strongly agree, 37 (36.3%) were – boys and 65 (63.7%) were girls. The study further reveals that there is a strong association between the sex of student and sharing information on their health with friends (chi-square value = 17.285, *p* < 0.001). Thus, the study ascertained that female students strongly share their information on health with friends than their male counterparts.

Table 7 further revealed that out of 42 respondents strongly disagreed that they share information on their health with friends, 16 (38.1%) were in Form two and 26 (61.9%) were in Form three. Out of 38 respondents who noted disagree, 18 (42.4%) were in Form two and 20 (52.6) were in Form three. From Table 7, out of 25 respondents who expressed neutral, 14 (56.0%) were in Form two and 11 (40.0%) were in Form three, and out of 184 respondents who indicated agree, 65 (35.3%) were in Form two and 119 (64.7%) were in Form three. In Table 7, out of 102 respondents who strongly agreed that they share information on their health with friends, 52 (51.0%) were in Form two and 50 (49.0%) were in Form three. The study also reveals that there is a significant association between the class of the student and sharing information on their health with friends (chi-square value = 19.258; *p* < 0.001). Thus, the study gives an indication that Form three students strongly share their information on health with friends than Form two students.

In furtherance, Table 7 shows that out of 105 respondents who strongly disagreed that they share information on their health with parents, 60 (57.1%) were boys and 45 (42.9%) were girls. From Table 7, out of 58 respondents who noted disagree, 32 (55.2%) were boys and 26 (44.8) were girls; and out of 55 of the respondents who expressed neutral, 18 (32.7%) were boys whereas 37 (67.3%) were girls. As shown in Table 7, out of 125 respondents who agreed, 50 (31.5%) were boys and 75 (68.5%) were girls. Again, out of 48 respondents who strongly agreed, 28 (58.3%) were boys and 20 (41.7%) were girls. The study reveals that there is a significant association between the sex of student and sharing information on their health with parents (chi-square value = 13.285, *p* = 0.004). Thus, the study gives an indication that female students share information on their health with their parents more than their male counterparts.

The results in Table 7 show that out of 105 respondents who strongly disagreed, 30 (28.6%) were in Form two and 75 (71.4%) were in Form three, and out of 58 respondents who noted disagree, 24 (41.4%) were in Form two and 34 (58.6%) were in Form three. From Table 7, out of 55 respondents who expressed neutral, 14 (25.5%) were in Form two and 41 (75.5%) were in Form three; out of 125 respondents who agreed, 68 (54.4%) were in Form two and 57 (45.6%) were in Form three. Table 7 shows that out of 48 respondents who noted strongly agree that they share information on their health with parents, 29 (60.4%) were in Form two and 19 (39.6%) were in Form three. The study found no significant association between the class of the student and sharing information on their health with parents (chi-square value = 9.527, *p* = 0.069).

Furthermore, Table 7 shows that out of 44 respondents who strongly disagreed that they share their information on health with the health professionals, 26 (59.1%) were boys and 18 (40.9%) were girls. Again, out of 49 respondents who noted disagree, 22 (44.9%) were boys and 27 (51.1%) were girls, and out of 38 respondents who expressed neutral, 16 (42.1%) were boys and 22 (57.9%) were girls. In addition, out of 145 respondents who indicated agree, 70 (48.3%) were boys and 75 (51.7%) were girls; out of 115 respondents who noted strongly agree, 82 (71.3%) were boys and 33 (28.7%) were girls who expressed that they share their information on health with health professionals. The study reveals that there is a strong significant association between the sex of student and sharing information on their health with health professionals (chi-square value = 17.987, *p* = 0.015). The implication of this is that female students share their information on health with the health professionals more than their male counterparts.

The results in Table 7 further show that out of 44 respondents who strongly disagreed, 16 (36.4%) were in Form two and 28 (63.6%) were in Form three; out of 49 of the respondents who noted disagree, 20 (40.8%) were in Form two and 29 (59.1%) were in Form three; out of 38 of the respondents who expressed neutral, 14 (36.8%) were in Form two and 24 (63.2%) were in Form three; out of 145 of the respondents who indicated agree, 60 (41.4%) were in Form two and 85 (58.6%) were in Form three; and out of 115 respondents who noted strongly agree, 55 (47.8%) were in Form two and 60 (52.2%) were in Form three, who noted that they share information on their health with health professionals. The study reveals that there is a significant association between the class of the student and sharing information on their health with health professionals (chi-square = 19.527, *p* < 0.001). The senior students were found to share their health problems with the health professionals.

From Table 7, out of 32 respondents who strongly disagreed that they share information on their health with school authorities, 18 (56.3%) were boys and 14 (43.8%) were girls; out of 52 of the respondents who noted disagree, 32 (61.5%) were boys and 20 (38.5) were girls; out of 42 of the respondents who expressed neutral, 24 (57.1%) were boys and 18 (42.9%) were girls. Again, from 155 of the respondents who indicated agree, 85 (54.8%) were boys and 70 (45.2%) were girls, and out of 110 respondents who noted strongly agree, 54 (49.1%) were boys and 56 (50.9%) were girls. The study did not find any significant association between the sex of students and sharing information on their health with school authorities (chi-square value = 16.231, *p* = 0.023).

Table 7 further revealed that out of 32 respondents who strongly disagreed for sharing information on their health with school authorities, 17 (53.1%) were in Form two and 15 (46.9%) were in Form three; from 52 of the respondents who noted disagree, 17 (32.7%) were in Form two and 35 (67.3%) were in Form three; out of 42 of the respondents who expressed neutral, 19 (44.5%) were in Form two and 23 (54.8%) were in Form three; from 155 of the respondents who indicated agree, 69 (44.5%) were in Form two and 86 (64.7%) were in Form three and out of 110 respondents who noted strongly agree, 43 (39.1%) were in Form two and 67 (60.9%) were in Form three. The study also reveals that there is a significant association between the class of students and sharing information on their health with school authorities (chi-square value = 18.258, *p* < 0.001). Thus, the study gives an indication that Form three students strongly share information on their health with school authorities than Form two students.

## Discussion

The study examines senior high school students' understanding and attitudes toward information on their health on campus in the Kumasi Metropolis. The study found that the students had fair knowledge about the causes and symptoms of malaria. This is not surprising as malaria is a staple disease in the tropical regions where the study area (Kumasi Metropolis) falls. In fact, according to the Ghana Health Service (13), malaria is the number one outpatient disease in Ghana which is likely to underpin the students' familiarity with the disease. Again, the students expressed enormous knowledge on the causes and symptoms of cholera. The students asserted that they experience an occasional outbreak of cholera on campus, particularly when they eat contaminated foods on campus. It is worthy to mention that the students' familiarity with the causes and symptoms of cholera may be as a result of the perennial outbreak of the cholera disease in the study areas. Some of the respondents attributed the outbreak of cholera to contaminated sources, foods and drinks sold by market vendors, heaped human waste, and undercooked food from the dining.

Again, the students showed fair knowledge about the causes and symptoms of the sexually transmitted diseases (gonorrhea and syphilis). The students' awareness about these diseases could be attributed to the fact that some aspects of these STIs are treated as part of their curriculum content at the junior high and senior school levels. Subjects such as integrated science and social studies contain some contents that bother on these diseases. Again, several adverts are run on radio and television concerning these diseases giving the students fair knowledge and understanding about the causes and symptoms of STIs (gonorrhea and syphilis). This is an indication that students in the metropolis have in-depth knowledge on the causes of STIs and this supports the study conducted by KHademian et al. (12), Waldman et al. (14), and Thanavanh et al. (2), which reveals that students have a high level of knowledge about the causes of STIs, particularly HIV or AIDS.

Moreover, the study found that most of the students in the Kumasi Metropolis do not use the internet to seek information on their health. This may be attributed to the reason that most of the schools do not have access to the internet in their respective schools and this impedes their accessibility to internet facilities to seek information on their health while on campus. Again, the students are not permitted to use phones in the school which further hinders their accessibility to the internet. However, the students who asserted that they seek information on their health from the internet claimed that they had high knowledge of their health. This contravenes the study of Hampton (8) that the people who most often seek information from the internet have not experienced any positive change in their lives.

On the other hand, the students were found to seek information on their health from health professionals in their schools. This is due to the fact that the selected schools are urban schools and have access to health facilities (sick bays) being operated by the health professionals on their campuses. This makes most of the students go to them to seek information concerning their health issues. This confirms a study by Chen et al. (2018) and Jordan et al. (3) that there is the need for health professionals to help and guide efforts by educating children and adults about their health information (issues) through more comprehensive tests. It is those health professionals who can detect one's health problems through a series of medical tests. The study further found that the students sought information on their health from their friends. The students expressed that they were comfortable in sharing information on their health with their friends because they believe their friends could keep the information to themselves without divulging it to a third party. Also, the students claimed that they incur costs when they consult the health professionals so they rather attend to their friends they perceive to be knowledgeable for help. However, they noted that the information they seek from their friends is sometimes not authentic.

In addition, the study found that the majority of the students do not seek information on their health from newsletters. This is because most of the students do not have access to newsletters on health while on campus. Even the library which was supposed to be a repository of these materials did not have them and the students barely sought information on their health for this source. However, the study found that the students sought information on their health from their textbooks. The students claimed that the information they seek are part of the things they learn in school. Such information includes the signs and symptoms of some common diseases such as malaria, STIs, and cholera. They further noted that they get some of the ways to treat certain diseases in their textbooks. Finally, the study found a strong association between the students' level of knowledge on the causes and symptoms of malaria, cholera, and STIs and the seeking information on their health from the internet, health professionals, and textbooks.

With respect to sharing information on their health by the students, the study found that most of the students in Kumasi Metropolis share their information with friends. The students claimed that their friends are always around them any time they are in need of help especially in schools and make them feel comfortable by sharing their information on health with them. They further asserted that they have confidence and trust in their friends that they will not share their information with a third party. The study further found a strong association between the sex of students and the sharing health information with friends. Female students were found more to be sharing information on their health with their friends than their male counterparts.

That notwithstanding, the study found that the students were hesitant in sharing information on their health with their parents. This may be attributed to the fact that most of the students are boarders in their respective schools, and as a result, they spend less time with their parents. It may also as a result of the bully nature of many Ghanaian parents. Because of the intimidating nature of some parents, the motherly or fatherly relationship between child and parents is marred, which culminates into students finding it difficult to share information on their health with their parents. This makes them keep information on their health to themselves as Sbaffi and Zhao (20) and Paterson (2010) noted that young people have a strong desire to be in control of their own record than sharing with intimidating parents.

However, the study reveals that the students in the Kumasi Metropolis share information on their health with the health professional in the school. The students asserted that they believe the health professionals keep the information confidential. This gives them some comfort and confidence to share information on their health with them. Again, it may also be attributed to the fact that the students believe in them to have the expertise to solve their health problems and therefore are willing to share information on their health with these professionals. This corroborates the study of Tran et al. (23), Ghana Health Service (13), and Teixeira et al. (35) that sharing information on their health with health professionals is hinged on their knowledge and their ability to keep the information confidential. Again, the sex of students was found to have a strong association with the sharing information on their health with health professionals. Female students were found to share information on their health with the health professionals more than their male counterparts. This may be precipitated by the Ghanaian culture where female students are mostly found to access health care whenever they fall sick as compared to their male counterparts who will keep their health problems to themselves.

## Conclusion and Policy Implications

The study examined senior high school students' understanding and attitude toward health information access in schools. The findings of the study have brought to the fore pertinent health issues that have been neglected in the educational sector for many decades, particularly in many developing countries such as Ghana. The study found students' knowledge of the causes and symptoms of malaria, cholera, and STIs to be appreciably high as a result of readings from textbooks and health professionals. Again, the study found that the students preferred sharing their health information with friends than their parents, particularly the female students. Again, the study found that the students felt uncomfortable sharing their health information with the schools' authorities for fear of stigmatization. Finally, the study further posits a strong association between the students' level of knowledge on the causes and symptoms of malaria, cholera, and STIs and the seeking health information from the internet, health professionals, and textbooks.

Following from the findings, the study recommends that the counseling units in the senior high schools should have a health professional in their team who would provide clinical counseling services to the students. Monitored positive peer-counseling group (this is where students are electronically and/or manually put into micro-groups of three in addition to a counseling expert for them to share their personal problems they feel to share hinged on the principle of trust and confidentiality) should also be encouraged by the schools' management and by extension the counseling units for the students to share views on themselves, particularly on health issues where they deem fit. In this case, the counseling expert would be able to moderate the conversations of the students and give expert advice appropriately. The study further proposes that schools' management should institute a telemedicine program in the schools to well-inform the students on health matters to prevent abuse of health information. Finally, the study recommends a strong health system on the campuses of senior high schools as they have become communities on their own as a result of the emergence of the free senior high school program.

## Limitations of the Study

Despite the innovative contributions of this study to the literature and policy of health information among students, certain limitations that might affect the generalizability and accuracy of findings were inevitable, particularly during the design and data collection phases of the study. Therefore, it is incumbent to note that the findings of this study should be interpreted in light of such limitations. For instance, given time and financial constraints, only five schools were selected for the study out of convenience. As a result, the extent to which the findings could be generalizable to other students elsewhere in Ghana may be limited. In furtherance, whereas longitudinal analysis may be desirable, this study employed a cross-sectional design as opposed to a longitudinal study. This may limit the determination of any causal and temporal relationships among the various outcomes and explanatory study variables. The findings should, therefore, be taken as associations rather than being causal. More so, in Ghana, age and certain health problems are often not openly reported owing to the associated stigma.

## Data Availability Statement

The raw data supporting the conclusions of this article will be made available by the authors, without undue reservation.

## Ethics Statement

The studies involving human participants were reviewed and approved by Committee on Human Research, Publication and Ethics of the School of Medical Sciences, Kwame Nkrumah University of Science and Technology/Komfo Anokye Teaching Hospital. Written informed consent to participate in this study was provided by the participants' legal guardian/next of kin. Written informed consent was obtained from the minor(s)' legal guardian/next of kin for the publication of any potentially identifiable images or data included in this article.

## Author Contributions

All authors listed have made a substantial, direct, and intellectual contribution to the work and approved it for publication.

## Conflict of Interest

The authors declare that the research was conducted in the absence of any commercial or financial relationships that could be construed as a potential conflict of interest.

## Publisher's Note

All claims expressed in this article are solely those of the authors and do not necessarily represent those of their affiliated organizations, or those of the publisher, the editors and the reviewers. Any product that may be evaluated in this article, or claim that may be made by its manufacturer, is not guaranteed or endorsed by the publisher.
